# Hyper-Branched Cyclodextrin-Based Polymers as Anticoagulant Agents: In Vitro and In Vivo Studies

**DOI:** 10.3390/bioengineering9120765

**Published:** 2022-12-04

**Authors:** Yousef Khazaei Monfared, Mohammad Mahmoudian, Gjylije Hoti, Daniel Mihai Bisericaru, Fabrizio Caldera, Roberta Cavalli, Parvin Zakeri-Milani, Adrián Matencio, Francesco Trotta

**Affiliations:** 1 Dipartimento di Chimica and NIS, Università di Torino, Via P. Giuria 7, 10125 Torino, Italy; 2 Faculty of Pharmacy, Tabriz University of Medical Sciences, Tabriz 5166-15731, Iran; 3 Dipartimento di Scienza e Tecnologia del Farmaco, Università di Torino, Via P. Giuria 9, 10125 Torino, Italy; 4 Liver and Gastrointestinal Diseases Research Centre and Faculty of Pharmacy, Tabriz University of Medical Sciences, Tabriz 5166-15731, Iran

**Keywords:** cyclodextrin, hyper-branched polymers, coagulation, calcium, EDTA, therapy, in vivo

## Abstract

This study tested the anticoagulant effect of cyclodextrin (CD) hyper-branched-based polymers (HBCD-Pols). These polymers were synthesized and tested for their coagulant characteristics in vitro and in vivo. Due to their polymeric structure and anionic nature, the polymers can chelate Ca^2+^, reducing the free quantity in blood. HBCD-Pol increased the blood clotting time, PT, and aPTT 3.5 times over the control, showing a better effect than even ethylenediaminetetraacetic acid (EDTA), as occured with recalcification time as well. A titration of HBCD-Pol and EDTA showed exciting differences in the ability to complex Ca^2+^ between both materials. Before executing in vivo studies, a hemocompatibility study was carried out with less than 5% red blood cell hemolysis. The fibrinogen consumption and bleeding time were analyzed in vivo. The fibrinogen was considerably decreased in the presence of HBCD-Pol in a higher grade than EDTA, while the bleeding time was longer with HBCD-Pols. The results demonstrate that the anticoagulant effect of this HBCD-Pol opens novel therapy possibilities due to the possible transport of drugs in this carrier. This would give combinatorial effects and a potential novel anticoagulant therapy with HBCD-Pol per se.

## 1. Introduction

Recent reports suggest thrombosis is responsible for one in four deaths worldwide, which is expected to increase due to the aging of the population. There are two types of thrombosis, arterial and venous; they are closely related to the existence of one another, and different factors, such as infections or diet, can influence their appearance. For example, they may be associated with cardiovascular diseases such as hyperlipidemia, smoking, or diabetes, which provoke the rupture of atherosclerotic plaques and promote the formation of a thrombus [1].

Antithrombotic agents are divided into three families: anticoagulants, anticoagulants and fibrinolytic agents. Another type is the anticoagulants that prevent fibrin formation, which predominates in venous thrombi. There are four FDA-approved anticoagulants: (i) heparins, which bind antithrombin [2], (ii) vitamin K antagonists (warfarin, [3]), (iii) direct thrombin inhibitors, and (iv) direct FXa inhibitors (Table 1). Typically, all current agents are associated with different secondary effects, such as bleeding. They can be oral or injected, although oral anticoagulants could present non-significant reductions in overall major bleeding, significantly lower rates of intracranial hemorrhage, and higher rates of gastrointestinal bleeding [4]. Due to all of the above, it has been continuously interesting to discover novel drugs with reduced adverse effects or carriers that increase the activity of the drug while decreasing the adverse effects. The increased bioavailability of drugs is an excellent option to achieve better results and safe profiles of drugs. In this field, the use of a carrier called cyclodextrin (CD) is highly considered.

CDs are well-known to the scientific community for their ability to solubilize poorly soluble drugs [6,7,8]. Chemically, CDs are cone-shaped oligosaccharides obtained from starch with α-(1,4) linked glucose units. The most common CDs are the natural derivatives with six, seven, and eight glucose units, α-, β- and γ-cyclodextrin (CD), respectively. The CD ring is a conical cylinder of an amphiphilic nature, with a hydrophilic outer layer (formed by the hydroxyl groups) and a lipophilic cavity [9]. Classically, poorly soluble drugs are complexed with CD, creating the so-called “inclusion complex”, which increases its solubility, stability, or bioactivity [10,11,12].

In some cases, their capacity lacks efficiency due to the drug’s complex structure or the desire for different profiles (e.g., slower release). Different materials have been prepared to improve their properties. This is the case of the hyper-branched CD-based polymers (HBCD-Pol, [5], Table 1), which are soluble but with a three-dimensional network and tunable structure with little/no toxicity [13]. Curiously, although free CDs did not present a coagulation effect [14], HBCD-Pol demonstrated the capacity to chelate different cationic ions, which could originate a combinatorial effect between the increase in activity of some anticoagulant for the complexation and the interferences with the Ca^2+^ signal [5,15].

Based on this last point, these intrinsic capacities for eventual use as an anticoagulant are presented in this research. This will open a gate for novel therapeutic approaches where HBCD-Pol can be administered with different drugs to control and manage the coagulation from a combinatorial point of view. In particular, different points will be studied in three different blocks:

(I) The capacity of HBCD-Pol to chelate Ca^2+^;
(II) The in vitro anticoagulant activity and its hemocompatibility to determine the blood clotting time and the plasma recalcification time. After these, we study the prothrombin time (PT) and the partial prothrombin time (PTT);
(III) The in vivo PT, PTT, fibrinogen, and bleeding time using rats as a model.

## 2. Materials and Methods

### 2.1. Materials

β-cyclodextrin (β-CD) was kindly provided by Roquette Freres (Lestrem–France). Pyromellitic anhydride (PMDA), dimethylsulfoxide (DMSO), triethylamine (Et_3_N), ethylenediaminetetraacetic acid (EDTA), thrombin from bovine plasma, partial thromboplastin time (aPTT) and prothrombin time (PT) assay agents were purchased from Sigma-Aldrich (Milan, Italy). Animal food was supplied by Javaneh Khorasan Co. (Iran). All chemicals and reagents used were of analytical grade unless otherwise specified. 

### 2.2. In Vitro Protocols and Experiments

#### 2.2.1. Synthesis of HBCD-Pol

Hyper-branched water-soluble β-CD polymer (HBCD-Pol) was prepared as reported [5]. Briefly, 6 mL of anhydrous DMSO and 1 mL of triethylamine were placed in a glass scintillation vial round-bottom flask and 0.997 g of β-CD was added until complete dissolution. Then, the required quantity of PMDA (pyromellitic dianhydride) was added to achieve a 1:12 (CD:linker) molar ratio, and the solution was allowed to react for 24 h at room temperature. Once the reaction was completed, the product was precipitated by adding ethyl acetate, and an excess of ethyl acetate was added during filtration to remove impurities. After drying, the product was solubilized in deionized water, lyophilized, and finally, residuals and unreacted reagents were completely removed by Soxhlet extraction with acetone for 24 h. Elemental analysis was used to confirm the total removal of DMSO. The white powder was dried and ground in a mortar, then preserved in dark and dry conditions. 

#### 2.2.2. Potentiometric Titration

The chelating agents used in potentiometric titration were HBCD-Pol and EDTA, forming chelates through the reaction with Ca^2+^. The titration was carried out according to the procedure described in the literature [16]. A 0.1 M NaOH solution, pre-standardized with 0.025 M oxalic acid solution, was used as a titrant. Titration was carried out using a volumetric manual burette by adding 0.1 mL of the titrant under gentle stirring of the analyte solution (50 mL) at room temperature. Subsequently, 500 mg of EDTA, HBCD-Pol and their complexes with 20 mg of Ca^2+^ ions were used as analytes. To reach an equilibrium between readings, it was necessary to add the titrant around every 60 s. pH values were continuously measured and recorded after each addition using a pH meter until a pH of 12 was reached. The titration curve of pH versus NaOH titration volume was generated, and the curve’s inflection point, using a second derivative method, was found for the indicated transition. Then, the volume of NaOH consumed at the inflection point is applied to the Equation (1) used for the calculation of the milliequivalents of acidity per 100 g of sample (m_eq_):

(1) $$\frac{m_{\mathrm{eq}}\mathrm{of}\;\mathrm{acidity}}{100\;g\;\mathrm{sample}}=\frac{V_{s}{*\;c}_{\mathrm{NaOH}}*100}{m_{s}}$$

where V_s_ is the volume of NaOH consumed by the sample, c_NaOH_ is the concentration of NaOH in mol/L and m_s_ is the mass of the sample [16].

#### 2.2.3. Anticoagulant Activity

Fresh Wistar male rat’s blood was used to evaluate the anticoagulant activity of HBCD-Pol compared to EDTA and control at room temperature without citrate sodium addition, according to the literature [17]. Different concentrations of 250, 500, 1000, 1500 and 2000 (µg/mL blood) of HBCD-Pol (5%) and concentrations of 500 and 1000 (µg/mL blood) for EDTA (5%) as a positive control were separately added to glass tubes with 1 mL of fresh rat blood. The blood that was free of additions was drawn to the tube as a control. Almost all blood samples were then checked over the next 1 and 3 h for detectable changes at room temperature, such as the formation of a clot.

#### 2.2.4. In Vitro Clotting Time

Blood clotting time (CBT) is a primary method to identify the prominent hemostatic agent [18]. Blood samples were collected from Wistar male rats and mixed with 3.8% sodium citrate. CBT was started by adding 1500, 1000 and 500 µg/mL of HBCD-Pol (5%) and the same concentrations for EDTA (5%) into a 5 mL glass tube, followed by incubating at 37 °C for 5 min, and the control group added nothing. Then, 1 mL of citrated rat blood was mixed with the samples and continued for incubation at 37 °C for 3 min. After that, 500 μL of 0.025 M CaCl_2_ aqueous solution was added into the tubes to trigger the coagulation pathway. The tubes were taken out of the water bath and inclined every 30 s until the blood in the tube did not flow. The clotting time was recorded as the result of CBT.

#### 2.2.5. Plasma Recalcification Time (PRT)

Plasma recalcification time (PRT) was determined according to the hook method [18]. Briefly, collected blood was mixed with 3.8% trisodium citrate (1/10 vol). Platelet-poor plasma (PPP) was obtained by centrifuging the fresh blood at 3000 rpm for 15 min at 4 °C. A fresh 300 μL of PPP was incubated at 37 °C with 1500, 1000 and 500 µg/mL blood of HBCD-Pol and the same amount for EDTA at tube tests for 3 min. The recalcification of plasma was checked using 30 μL of 0.5 M CaCl_2_. Plasma was observed every 1 min to estimate fibrin thread formation in PPP. PRT was designated as the time to recalcification from the time of the addition of CaCl_2_. 

#### 2.2.6. Partial Thromboplastin Time (aPTT) and Prothrombin Time (PT) Assays

All the test samples, test reagents and CaCl_2_ solution were incubated at 37 °C in advance [18,19]. To test aPTT, 100 µL of PPP and 100 µL of aPTT reagent were incubated at 37 °C for 5 min, and then different concentrations (1500, 1000 and 500 µg/mL blood) of HBCD-Pol 5% and the same volume of EDTA 5% was added. Then, 100 µL of CaCl_2_ 0.025 M was added to the tubes. The prothrombin time (PT) test was performed by adding 100 µL of PPP, which was mixed with both HBCD-Pol and EDTA 5% as the same used concentrations for aPTT and then 200 µL of PT reagents to the tube successively. The negative controls were considered pure aPTT and PT reagents without samples. The PTT and PT were tested with an automatic coagulometer (ACL 300R, Instrumentation Laboratory).

#### 2.2.7. Hemocompatibility Test

The hemolytic activity of HBCD-Pol was evaluated according to the standard technique [20]. The freshly citrated prepared blood samples were collected and centrifuged at 1000 rpm for 5 min. Then, the plasma was removed, and approximately 5 mL of sterile phosphate-buffered saline (PBS), pH 7.4, was added with repeated centrifugation at 1000 rpm for 5 min to remove the plasma residues. Then, 1000 µL of HBCD-Pol were mixed with blood tubes and incubated at 37 °C and 150 RPM for 90 min. After incubation, the tubes were placed on an ice bath for 5 min and then centrifuged at 1000 rpm for 5 min. Then, 100 µL of supernatant was removed from the tube and then diluted with a refrigerated PBS solution to yield 2% *v*/*v*. The same procedure was repeated with PBS as a negative control and Triton X-100 (0.1% by volume) as a positive control. Finally, each sample’s absorbance was calculated at a value of 540 nm. 

### 2.3. In Vivo Experiments

For in vivo experiments’ published protocols with modifications were used [21]. Briefly, BALB/c male mice weighing 20–25 g were used. The HBCD-Pol and EDTA were administered intraperitoneally (i.p.) in a fixed volume of 200 μL in three dosages, 40, 20 and 10 mg/kg, for both agents. In each experimental session, at least five animals per treatment group were tested; control groups were run at the beginning and at the end of every experimental session. Mice were accustomed to handling by the investigators, and the injections were carried out by skilled investigators with minimal disturbance to the animals. The experiments were approved by the Tabriz University of Medical Sciences Pharmacy Department under the Iran Ethical Code: IR.TBZMED.AEC.1401.022.

#### 2.3.1. APPT and PT Assays

Blood was collected from ether-anesthetized mice by cardiac puncture and anticoagulated with 3.8% trisodium citrate (1/10 vol) of mice treated by HBCD-Pol and EDTA at the determined dosage 10 min after injection. Anticoagulated blood was immediately centrifuged for 5 min at 12,000× *g*, and the supernatant platelet-poor plasma (PPP) was separated and transferred onto melting ice until tested (generally within 1 h) or frozen at –80 °C. Both activities were measured by standard assays, as mentioned in Section 2.2.6.

#### 2.3.2. Fibrinogen Measurement

Blood was collected from ether-anesthetized BALB/c mice by cardiac puncture and anticoagulated with trisodium citrate (1/10 vol) of mice treated with HBCD-Pol and EDTA at a determined dosage 2 min before the thrombotic challenge, which was induced by the i.p. injection of bovine thrombin. The dose of thrombin used was selected as 1000 U/kg [22]. Plasma fibrinogen was measured by the Clauss method in a Coagulab MJ coagulometer (Ortho Diagnostics) using bovine thrombin. 

#### 2.3.3. Bleeding Time

Bleeding time was assessed by a tail transection method [22,23]. Briefly, BALB/c mice were treated with HBCD-Pol and EDTA at 40 m/kg dosage; this dosage was selected according to the results of our previously mentioned experiments for 10 min. Then, the mice were positioned in a special immobilization cage that kept the tail steady and immersed in saline thermostated at 37 °C. After 2 min, the tip of the tail was transected with a razor blade at approximately 2.5 mm from its end. The tail was immediately reimbursed in warm saline, and the bleeding time was recorded. The endpoint was the arrest of bleeding lasting for more than 30 s.

## 3. Results and Discussion

### 3.1. Potentiometric Titration

As mentioned above, the capacity of HBCD-Pol to chelate metals was previously described [5]. However, it is crucial to determine the interaction ability of the synthesized polymer. Ethylenediaminetetraacetic acid (EDTA) is a well-known chelating agent used in several industries to determine Ca^2+^, although its activity can be affected by other ions, such as Na^+^ [24,25]. 

**Figure 1:**
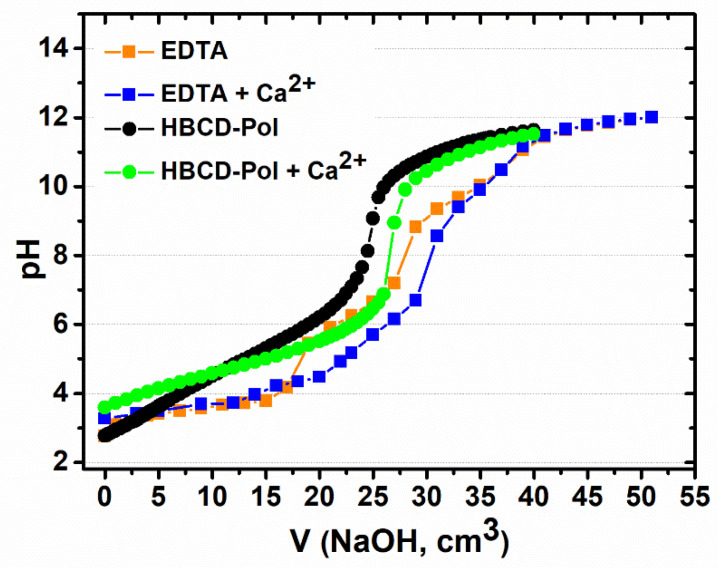
Potentiometric titration curves of EDTA, HBCD-Pol, and their complexes with Ca^2+^.

Figure 1 presents the delay on the equivalent point of EDTA compared to HBCD-Pol. The results suggest the more robust binding sites of EDTA that are characterized by 581.6 m_eq_ of acidity, compared to HBCD-Pol with 461.1 m_eq_ of acidity. It is important to mention that near the physiological and blood pH, the volumes of NaOH are ~25 mL for HBCD-Pol and ~31 mL for EDTA. Therefore, the higher the presence of COOH groups in the solution, the higher the interaction, and the volume of NaOH required to neutralize the compound (the equivalence point) is also high.

The results demonstrated (i) a higher chelating strength of EDTA in comparison with HBCD-Pol, with closer values at physiological pH; and (ii) the presence of Ca^2+^ affects both compounds. It is reasonable to have a lower effect of HBCD-Pol than EDTA against Ca^2+^. However, the effect on global coagulation is affected by several factors, not only Ca^2+^, such as the presence of other molecules that can be complexed by HBCD-Pol, affecting the global coagulation profile.

### 3.2. In-Vitro Anticoagulant Study

**Figure 2:**
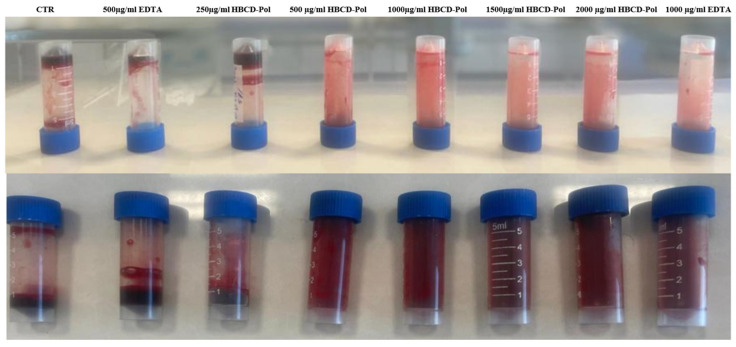
Anticoagulant activities of HBCD-Pol and EDTA after 3 h.

The anticoagulant activity was evaluated by adding the HBCD-Pol and EDTA to rat blood samples at different concentrations. Figure 2 shows the results, where the control sample without addition, HBCD-Pol (250 µg/mL) and EDTA (500 µg/mL) initiated coagulation after 15, 20 and 18 min of incubation, respectively. In addition, the thickened blood samples were checked after 60 min to find a large clot in the tubes. Surprisingly, after one hour, there were no signs of clotting in the HBCD-Pol and EDTA at 500 and 1000 µg/mL, but after three hours, only some clotting was observed for both of them, although these clots did not prevent blood flow. 

The results suggest that HBCD-Pol could present a higher anticoagulation effect than EDTA. Therefore, to better understand its ability, the in vitro clotting time (CT) using fresh blood clotting time was carried out according to the literature [2]. Blood samples were collected and mixed with 3.8% sodium citrate as an anticoagulant, and then the samples were warmed to 37 °C in a water bath and added to 20 μL of 0.2 M CaCl_2_. The CT was designated as the time when no flow of samples was observed after the tubes were inverted.

**Figure 3:**
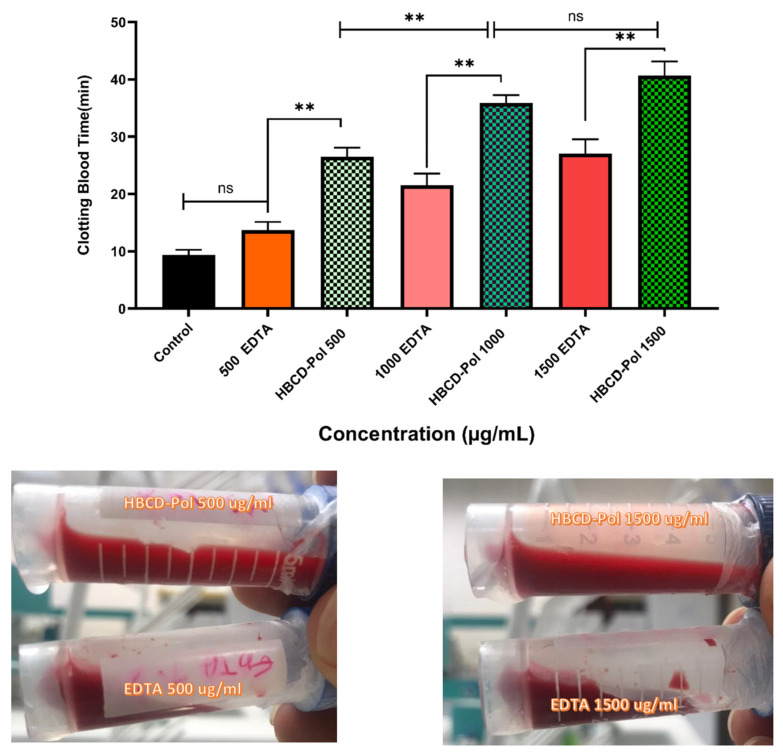
Blood clotting time assessment on different concentrations of HBCD-Pol and EDTA (5%). ** *p* < 0.01 and ns (not significant).

Interestingly, the results showed that the time of clotting was significantly increased by about two times by HBCD-Pol at different concentrations when compared to EDTA-K3 as the positive control (*p* < 0.01). In contrast, EDTA at 500 µg/mL could not show a difference from the control (Figure 3). 

**Figure 4:**
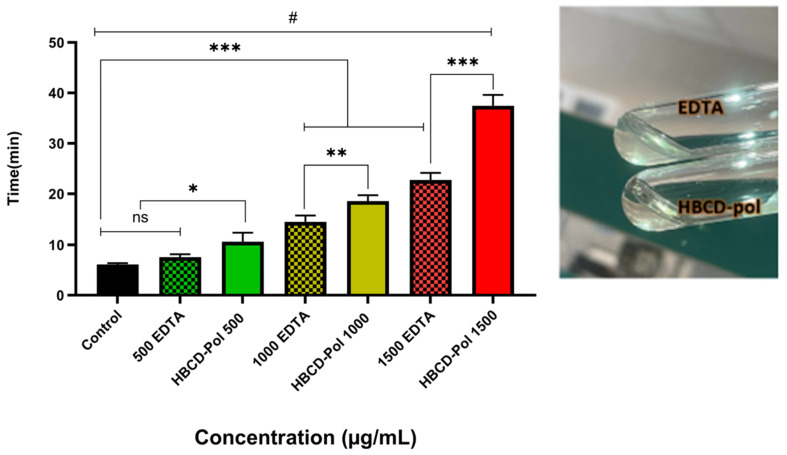
Plasma recalcification time on platelet-poor plasma for different amounts of HBCD-Pol and EDTA. # *p* < 0.0001, *** *p* < 0.001, ** *p* < 0.01, * *p* < 0.05 and ns (not significant).

In the next step, the plasma recalcification time parameter was evaluated on platelet-poor plasma (PPP) at three different concentrations for both HBCD-Pol and EDTA. Surprisingly, the results showed that adding HBCD-Pol into plasma at 1500 g/mL led to a considerable increase in clotting time of about 4 and 2 times compared to control and EDTA at that concentration, respectively, while the EDTA at 500 g/mL did not show differences compared to the control. Meanwhile, HBCD-Pol at other concentrations showed a better ability to enhance the clotting time compared to EDTA at the same amount (Figure 4). 

### 3.3. PT and aPTT Tests

**Figure 5:**
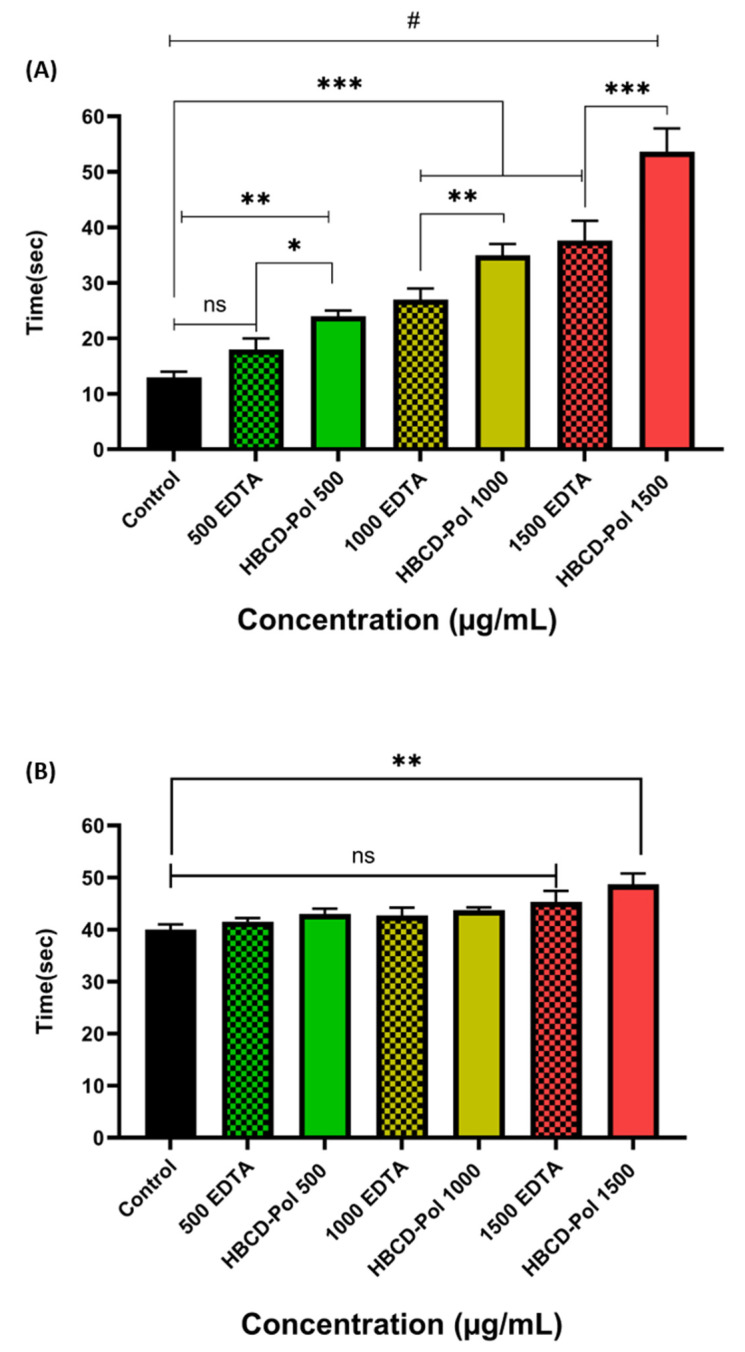
(**A**) aPTT and (**B**) PT assessment at various concentrations. # *p* < 0. 0001, *** *p* < 0.001, ** *p* < 0.01, * *p* < 0.05 and ns (not significant).

The next step in the study was to evaluate the activated partial thromboplastin time (aPTT) and prothrombin time (PT) [7,8]. The pre-warmed PPP, aPTT and PT reagents were incubated at 37 °C for 5 min with HBCD-Pol 5% and EDTA 5% at various concentrations. Then, the reactions were started by adding the CaCl_2_ 0.025 M to the tubes. The results showed that HBCD-Pol and EDTA could not affect the aPTT parameter except at 1500 g/mL (*p* < 0.01). HBCD-Pol illustrated a much better effect at all concentrations, especially at 1500 g/mL (*p* < 0.001), regarding increasing the PT parameter compared to EDTA. Indeed, the outcomes of this experiment confirmed that HBCD-Pol may be able to inhibit the extrinsic and common coagulation pathway because of its effect on increasing the PT time (Figure 5). In the bibliography, it is indicated that concentrations of α-, β- and γ-CD of 0.5% did not show any aPTT or PT variation [14]. However, the polymer above 0.05% showed differences, possibly due to the combination of Ca^+2^ chelation and the more complex structure and complexation capacities of the polymer with some coagulation factors. 

### 3.4. Hemocompatibility

**Figure 6:**
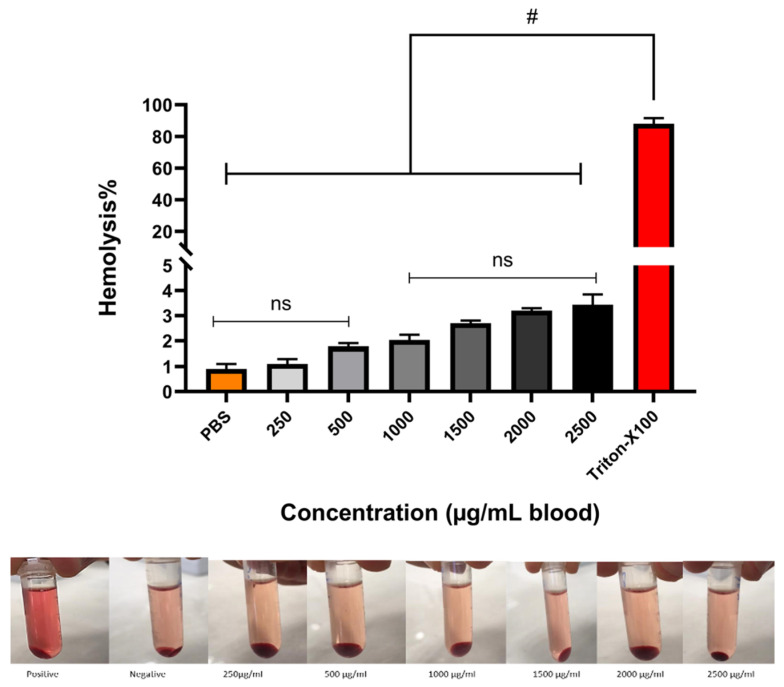
Hemolysis percentages of RBCs at 250–2500 μg/mL of HBCD-Pol free. PBS and Triton X-100 were used as the negative and positive control, respectively. # *p* < 0.0001 and ns (not significant).

The hemocompatibility of the HBCD-Pol was tested at different concentrations as a crucial parameter to use in an in-vivo experiment [9,10]. The hemolysis activity was studied in order to prevent hemolysis, thrombosis and embolization [11]. The hemocompatibility performance was shown in Figure 6, with a value of about 3% for both 2500 and 2000 µg/mL concentrations, while other concentrations revealed less than 2.5% hemolysis. All samples can be characterized as hemocompatible according to ISO 10993 (<5%) [12]. It is important to mention that α- and β-CD showed toxicity (up to 40%) at concentrations around 1% [14], while the polymer around 0.25% (2500 µg/mL) not only obtained no toxicity but also showed an effect on coagulation.

### 3.5. In Vivo Anticoagulant Characterization 

#### 3.5.1. Effects of Anticoagulation Agents on Clinical Coagulation Parameters

**Figure 7:**
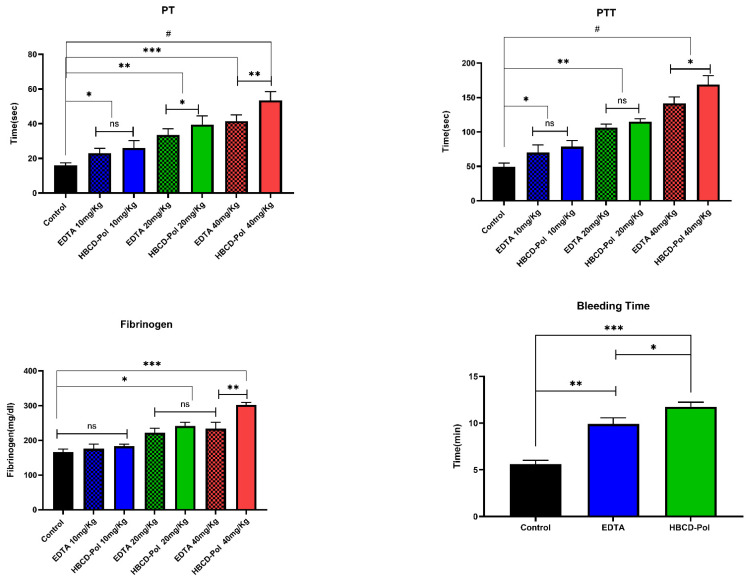
PT and aPTT in mice treated with HBCD-Pol and EDTA. Fibrinogen levels in mice injected with thrombin (1000 U/kg) after pretreatment and the tail transection bleeding time of animals pretreated. Data are the mean ± SEM (five animals for each group). # *p* < 0.0001, *** *p* < 0.001, ** *p* < 0.01, * *p* < 0.05 and ns (not significant).

Using a mouse model, the anticoagulant effects of HBCD-Pol and EDTA with thrombin were assessed. We selected a maximum dose of 40 mg/kg without visual signs of toxicity. aPTT and PT assays on samples collected 10 min after the agents’ injection showed a dose-dependent prolongation of aPTT and PT (Figure 7). However, doses corresponding to 40 mg/kg of HBCD-Pol showed the highest level of prolonged aPTT (>3.5 fold than control), while EDTA at that dose (40 mg/kg) caused a prolongation less than HBCD-Pol (*p* < 0.05). In addition, there were no significant differences for other dosages between the HBCD-Pol and EDTA. Interestingly, no increase in PTT in the in vitro experiment was observed (Figure 5). Furthermore, the results of the CT test showed a significant effect of HBCD-Pol to increase the plasma clotting time more than three times compared to control at the highest dosage. Moreover, HBCD-Pol could show a better anticoagulation effect at that dosage (40 mg/kg) when compared to EDTA (*p* < 0.01), while this difference was not seen at lower dosages (Figure 7). These results support the idea of a combinatorial effect due to the complexation of some coagulant agents, which decreased the effectivity of the pathway.

#### 3.5.2. Fibrinogen Consumption and Bleeding Time Test

CDs have a weak impact on fibrinogen’s microenvironment but can slightly affect its activity [14].Therefore, this section evaluated the capability of HBCD-Pol and EDTA to prevent fibrinogen consumption as an in vivo measure of thrombin inhibition. Mice were treated with HBCD-Pol and EDTA at the same dosages that were used for in vivo PT and PTT tests. Then, the plasma fibrinogen was determined on samples taken 2 min after the thrombin challenge. Both HBCD-Pol and EDTA at the highest dosage (40 mg/kg) effectively attenuated fibrinogen consumption by preserving about 45% and 30% of the circulating protein, respectively (Figure 7). On the contrary, the 10 mg/kg dosage showed a clearly weaker effect. The difference in fibrinogen levels between HBCD-Pol and EDTA-treated animals at 40 mg/kg was statistically significant (*p* < 0.01). 

**Figure 8:**
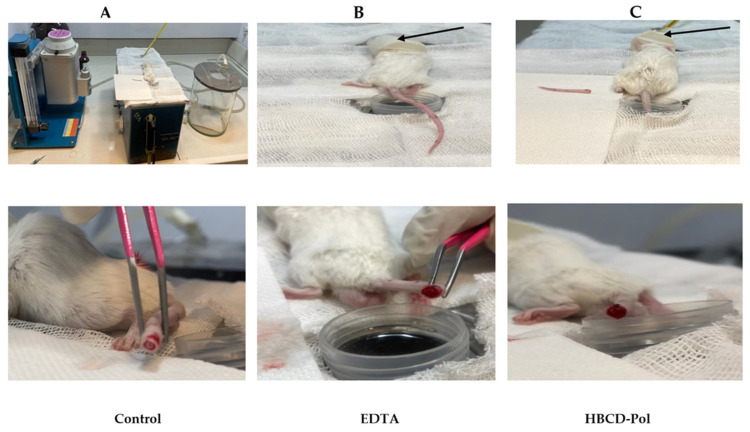
(**A**) including nebulizer machine, water bath, anesthesia induction chamber, left to right, respectively; (**B**,**C**), the tail transection procedure. The results of bleeding time in vivo experiment after 6 min of tail cutting. Small anesthesia chamber indicated with an arrow (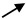).

The tail transection bleeding time was measured in animals receiving the dosage of 40 mg/kg of each agent 10 min after treatment. In line with the aPTT results, the bleeding time was significantly more prolonged in mice treated with HBCD-Pol and EDTA (2- and 1.7-fold increase, respectively) than in the control group mice (Figure 7 and Figure 8).

As a whole, these results suggest that HBCD-Pol increased the slight effect on the coagulation of natural CDs. Although it is true that its Ca^2+^ chelation capacity is lower than EDTA at the studied conditions, a higher in vitro and in vivo effect is obtained. Between both intrinsic (aPTT) and extrinsic (PT) pathways, the most affected is the intrinsic pathway. This pathway depends on more coagulation factors than the extrinsic pathway. The importance of Ca^2+^ in both pathways is evident, as suggested in Figure 5A or Figure 7, as in both cases, this is the pathway more affected. However, EDTA does not present a possible mechanism than HBCD-Pol yes: firstly, HBCD-Pol is able to chelate Ca^2+^, possibly creating an environment where [Ca^2+^]_app_ is lower, avoiding coagulation. Secondly, the polymeric structure could interact with the different factors of the pathway. Although natural CDs do not affect the aPPT pathway [14], the additional cavities where non-typical cyclodextrin guest can be complexed could increase the interaction and prevent the continuation of the pathway [26]. For example, the low interaction of fibrinogen with CDs could be increased for HBCD-Pol, boosting the effect of the material in coagulation [14].

## 4. Conclusions

To conclude, in this study, the anticoagulant effect of HBCD-Pol, a CD-based polymer was analyzed in vitro and in vivo. Due to its polymeric structure and anionic nature, the polymer can form a chelate with Ca^2+^ in vitro but with a lower strength than EDTA. However, HBCD-Pol increases the CT, PT and aPTT 3.5 times over the control, showing a better effect than even EDTA, as occurs with recalcification time, as well. The hemocompatibility test showed better results than EDTA and CD values reported in the bibliography. The fibrinogen consumption and bleeding time were analyzed in vivo; in all cases, the effect of HBCD-Pol at the same concentration of EDTA was higher. The results demonstrate that the anticoagulant effect of this HBCD-Pol could be used as a novel therapy from a combinatorial point of view: adding a drug at the same time as the polymer.

## Figures and Tables

**Table 1:** Examples of anticoagulant drugs in comparison with EDTA and HBCD-Pol.

	Anticoagulant Drugs		Molecule	Polymer
	Enoxaparin (synthetic heparin) ɫ	Warfarin ɫ	EDTA ɫ	HBCD-Pol [5]
Schematic structure	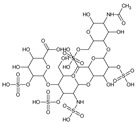	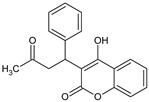	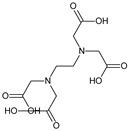	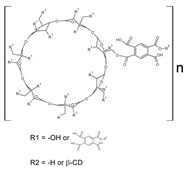
Description	Synthetic heparin, drug	Synthetic anticoagulant	Chelant	Polymer
Molecular formula	C26H42N2O37S5	C19H16O4	C10H16N2O8	(C162H94o107)n
Molecular weight	1134.9	308.3	292.24	37–42Kda
Solubility (mg/mL)	50	17 × 10^−3^	1 × 10^3^	800
Bioactivity	Drug similar to that of heparin (binds to antithrombin, AT), although it exhibits a higher ratio of anti-Factor Xa to anti-Factor Iia activity	Drug; inhibits the regeneration of vitamin K1 epoxide and thus the synthesis of vitamin K-dependent clotting factors, which include Factors II, VII, IX and X, and the anticoagulant proteins C and S	Chelant; induces anticoagulation by Ca^2+^ binding	Aim of present work
Can vehiculize biomolecules?	-	-	-	Yes

ɫ extracted from PUBCHEM (CID 772, 54678486 and 6049 on 23 November 2022).

## Data Availability

Not applicable.
